# An Error Corrected

**Published:** 1875-10

**Authors:** 


*An Error Corrected.*

The last graduating class of the Medical Department of the Nashville and Vanderbilt Universities numbered seventy-one, instead of fifty-eight, as has been stated by some of our exchanges. We regard, however, this correction of little importance, compared with the clinical advantages this institution will be prepared to give in future, as will be seen by the following editorial which we clip from the *Nashville Medical Journal*:

"The progress of time has developed one fact in relation to medical teaching, which cannot be ignored by any faculty or board of trustees—namely, that clinical teaching is an absolute necessity. Every medical college which cannot supply an abundance of clinical material will certainly fail to receive patronage sufficient to sustain it, and will have to close its doors sooner or later.

"To be really profitable, clinical instruction should be at the bedside of the patient, and in order that this shall be done hospitals should be directly connected with medical colleges.

"The Faculty of the Medical Department of the University of Nashville and the Vanderbilt University, feeling the importance of increased facilities for clinical instruction, determined, a few months since, to erect a large hospital on the college grounds, in in direct connection with the college building.

"The hospital building is situated on College street, with a frontage of one hundred and fifty-six feet, the central portion being three stories, and the wings (one on each side of the central portion) two stories, with a basement. The front of the building is ornamented with a porch fifty by ten feet, from the upper stories of which may be obtained the most picturesque view of the city and its beautiful surroundings. The central building is intended for the main entrance and the executive department, dispensary, office, library, etc. On each side of this executive department are two wards, fifty-two by twenty-eight feet, and fifteen feet high to ceiling. Each of the four wards is ventilated by ten large windows, flues in the walls, and by a stack ventilator. They are freely supplied with hot and cold water, and brilliantly lighted with gas. Each ward has attached, water-closet, bath-room, linen-room, dead-room, etc.

"Besides the larger wards, there are several smaller, for venereal patients, for lying-in women, etc., and others still for patients who are able to pay their expenses. All the wards communicate directly with a large clinical lecture-room, into which patients are conveyed on a new and improved hospital railway.

"The large *campus* around the building is to be ornamented with shade-trees, serpentine walks, flowers, arbors, fountains, etc., for the benefit of convalescing patients. It is, in fact, to be complete in all its appointments.

"The buildings will be completed and ready for occupancy in two weeks from this writing.

"Dr. A. A. East, recently assistant physician to the Tennessee Insane Asylum, has been elected physician and superintendent, and will furnish the buildings and enter upon the discharge of his duties as soon as the painters finish their work. We think Dr. East is peculiarly adapted to the position. He was a favorite student of ours; and after serving through the war as a surgeon, he was appointed first assistant physician to the lunatic asylum, a position which he filled with great credit to himself and benefit to that institution, for ten years. He is thoroughly conversant with the details of hospital service.

"Nashville has, for a long time, been a surgical center for a large extent of country; and the surgical clinic at our medical college has been unsurpassed. More than two hundred surgical cases were presented to the last class, involving operations of every magnitude, from amputation at the hip-joint to avulsion of the toe-nail.

"Now that preparations have been made to bring patients before the class—or, rather, to bring the class before the patient at the bedside—no better field for medical clinics can be found by the student seeking a medical education."

This institution can now be numbered among those that we endorse, and allowed a place in our advertising department. P.

